# Pumping Iron in Australia: Prevalence, Trends and Sociodemographic Correlates of Muscle Strengthening Activity Participation from a National Sample of 195,926 Adults

**DOI:** 10.1371/journal.pone.0153225

**Published:** 2016-04-27

**Authors:** Jason A. Bennie, Zeljko Pedisic, Jannique G. Z. van Uffelen, Melanie J. Charity, Jack T. Harvey, Lauren K. Banting, Ineke Vergeer, Stuart J. H. Biddle, Rochelle M. Eime

**Affiliations:** 1 Active Living & Public Health Program, Institute of Sport, Exercise and Active Living (ISEAL), Victoria University, Melbourne, Victoria, Australia; 2 Federation University Australia, Faculty of Health, Ballarat, Victoria, Australia; University of Sydney, AUSTRALIA

## Abstract

**Objective:**

The current Australian Physical Activity Guidelines recommend that adults engage in regular muscle-strengthening activity (e.g. strength or resistance training). However, public health surveillance studies describing the patterns and trends of population-level muscle-strengthening activity participation are sparse. The aim of this study is to examine the prevalence, trends and sociodemographic correlates of muscle-strengthening activity participation in a national-representative sample of Australians aged 15 years and over.

**Methods:**

Between 2001 and 2010, quarterly cross-sectional national telephone surveys were conducted as part of the Australian Sports Commission's 'Exercise, Recreation and Sport Survey'. Pooled population-weighted proportions were calculated for reporting: [i] no muscle-strengthening activity; [ii] insufficient muscle-strengthening activity, and [iii] sufficient muscle-strengthening activity. Associations with sociodemographic variables were assessed using multiple logistic regression analyses.

**Results:**

Out of 195,926 participants, aged 15–98 years, only 10.4% (95% CI: 10.1–10.7) and 9.3% (95% CI: 9.1–9.5) met the muscle-strengthening activity recommendations in the past two weeks and in the past year, respectively. Older adults (50+ years), and those living in socioeconomically disadvantaged, outer regional/remote areas and with lower education were less likely to report sufficient muscle-strengthening activity (p<0.001). Over the 10-year monitoring period, there was a significant increase in the prevalence of sufficient muscle-strengthening activity (6.4% to 12.0%, p-value for linear trend <0.001).

**Conclusions:**

A vast majority of Australian adults did not engage in sufficient muscle-strengthening activity. There is a need for public health strategies to support participation in muscle-strengthening activity in this population. Such strategies should target older and lower educated adults, and those living in socioeconomically disadvantaged, outer regional/remote and areas.

## Introduction

The prevention of chronic diseases, such as cardiovascular disease, diabetes, breast and colon cancer is a leading Australian [1], and global public health challenge [2]. Insufficient physical activity is among the leading and potentially preventable causes of chronic disease [3]. For chronic disease prevention, the Australian Department of Health and the World Health Organization recommend that adults should participate in [i] ≥150 minutes/week of moderate-to-vigorous-intensity aerobic activity (e.g. brisk walking or jogging) and, [ii] muscle-strengthening activities involving major muscle groups on two or more days per week [3, 4].

The 2014 Physical Activity and Sedentary Behaviour Guidelines were the first Australian national guidelines to recommend muscle-strengthening activities for adults and older adults [4]. Muscle-strengthening activities are performed with the primary purpose of increasing muscle strength or tone [5], and include activities such as lifting weights (e.g. barbells or dumbbells), and using resistance bands or body weight (e.g. push-ups, sit-ups) [6]. Exercise trials and epidemiological studies have shown that muscle-strengthening activities are associated with multiple favourable health outcomes, including improved metabolic [7–9], musculoskeletal, functional and mental health-related outcomes [10], improved blood lipid profile [11], and reduced blood pressure [12].

Despite these health benefits, few studies have reported the prevalence and correlates of muscle-strengthening activity participation [6]. Previous studies from US [13–15], Australia [16–18] and Japan [19] suggest a wide range of participation rates, with between 4% and 32% of adults (aged 18 years and over) meeting the muscle-strengthening activity recommendations of at least 2 sessions/week. While little is known about the sociodemographic correlates of participation in muscle-strengthening activity, there is some evidence that males, younger adults (18–24 years) and those with higher education levels are more likely to meet the recommendations [6]. Developing an understanding of the prevalence and correlates of muscle-strengthening activities is vital for orientating public health approaches to promote this essential component of physical activity-related health [6]. However, at present, only one study has examined the patterns and sociodemographic correlates of muscle-strengthening activity among a representative population-based sample of Australians [16], and none have examined its time trends.,.

The aim of this study was, therefore, to examine the prevalence, trends and sociodemographic correlates of muscle-strengthening activity among a large national-representative sample of Australians aged 15 years and over.

## Materials and Methods

### Sample

Data were used from the Australian Sports Commission’s ‘Exercise, Recreation and Sport Survey (ERASS)’ [20]. The ERASS entailed a series of yearly independent cross-sectional national surveys conducted between 2001 and 2010, with the aim of collecting information on the frequency, duration, and type of activities Australians participated in for exercise, recreation or sport [20]. The applicability of ERASS for use in public health surveillance has been established previously [21]. During 2001–10, quarterly survey samples were randomly selected from community-dwelling persons aged 15 years and over. Data were collected using a computer-assisted telephone interview (CATI) system with households being sampled from the Electronic White Pages (2001–2006) or by Random Digit Dialling technique (2007–2010) [20]. Data collection was commissioned by Australian Sports Commission to be carried out by an external market research company [20]. Upon being contacted by telephone, participants were provided with an an explanation of the purpose of the ERRAS via the use of a standardised text. Participants were explained that verbal consent was considered if they agreed to then take part in the telephone interview. Given the nature of the study, written consent was considered not practical. The market research company responsible for data collection recorded participant consent using a standardised data collection form. All study materials were securely stored by the company. At the commencement of each data collection period the Australian Sports Commission were provided with all data collection forms accrued during the study. Ethical approval for all procedures implemented in the ERRAS was obtained from Federation University Australia Ethics Committee. Response rates were 49.0%, 48.0%, 45.3%, 41.0%, 34.0%, 42.0%, 31.4%, 25.7%, 25.2% and 23.1%, from 2001 through 2010 respectively [20]. More details on ERASS methods can be found elsewhere [22, 23].

### ERASS questionnaire

The ERASS 2001–10 questionnaire asked respondents about participation in leisure-time physical activity, defined as; ‘*any physical activity done for exercise*, *recreation or sport in the past 12 months’*. Respondents were asked to exclude *‘any physical activity associated with work*, *household or garden chores’*. Those who indicated participation were asked to list the types of leisure-time physical activity undertaken, whether each activity was organised or non-organised, and the number of times they participated in each activity during the previous 12 months. From 2005 onwards, participants were also asked about the frequency and average session duration in the past two weeks. In the current study, we used data from the 12-month (2001–10) and 2-week recall periods (2005–10).

During ERASS data collection, respondents reported 187 different types of leisure-time physical activities that were initially collapsed into 170 categories [20]. In the current study, we considered leisure-time physical activities that are theoretically related to muscle-strengthening and were either organised or non-organised. A total of nine leisure-time physical activities were classified as primarily muscle-strengthening activities: *(i) ‘Calisthenics’; (ii) ‘Gymnasium workouts’; (iii) ‘Military exercise’; (iv) ‘Prime movers (over 50s)’; (v) ‘Body building’; (vi) ‘Circuits’; (vii) ‘Power team’; (viii) ‘Weight training for fitness’; and (ix) ‘Weightlifting (competition)*’. Responses from these activities were collapsed to create the overall muscle-strengthening activity variable. Similar questions on muscle-strengthening activities have been shown to have acceptable reliability [24], and have been used in previous Australian studies [17, 25].

### Classification of muscle-strengthening activity levels

Muscle-strengthening activity data from the 12-month recall were categorised as: [i] ‘no muscle-strengthening activity’ (0 sessions), [ii] ‘insufficient muscle-strengthening activity’ (1–103 sessions), and [iii] ‘sufficient muscle-strengthening activity’ (≥104 sessions). Data from the 2-week recall were classified as: [i] ‘no muscle-strengthening activity’ (0 sessions), [ii] ‘insufficient muscle-strengthening activity’ (1–3 sessions), and [iii] ‘sufficient muscle-strengthening activity’ (≥4 sessions). The ‘sufficient muscle-strengthening activity’ group (≥4 sessions) was further dichotomised according to the total duration of muscle-strengthening activities in the past two weeks into: [i] ‘sufficient frequency of muscle-strengthening activity’ (≥4 sessions and <80 minutes), and [ii] sufficient frequency and duration of muscle-strengthening activity (≥4 sessions and ≥80 minutes). The Australian Physical Activity Guidelines do not specify the recommended duration of muscle-strengthening activities; hence the cut-off point of 80 minutes in the past two weeks was used in accordance with a previous Australian study [18]. The duration was taken into account only for 2-week recalls, because no such data were available for 12-month recalls.

### Sociodemographic variables

Sociodemographic variables (gender, age, level of education) were assessed using standard questions [20], and sub-categories were created consistent with previous studies reporting on the ERASS (Table 1) [22, 26]. Participants’ state and postcode, were used to describe the distribution of the sample by Australian states and territories, area of residence [27] and regional socioeconomic status using the Australian Bureau of Statistics (ABS) ‘Index of Relative Socio-Economic Advantage and Disadvantage’ (IRSAD) [28]. IRSAD scores were grouped as quintiles, with ‘1’ for the most disadvantaged and ‘5’ for the least disadvantaged [28].

**Table 1:** Sample size and percentage (%) distribution^a^ of Exercise, Recreation and Sport Survey (ERASS) 2001–10 respondents—overall and by sociodemographic characteristics.

	2001	2002	2003	2004	2005	2006	2007	2008	2009	2010	Pooled (2001–10)
	*n*										
**Total sample**	*15*,*477*	*17*,*325*	*17*,*341*	*17*,*299*	*21*,*149*	*23*,*226*	*20*,*430*	*21*,*045*	*21*,*031*	*21*,*603*	*195*,*926*
	*n (%)*										
**Gender**											
Males	7,665 (49.5)	8,585 (49.6)	8,594 (49.6)	8,586 (49.6)	10,513 (49.7)	11,410 (49.1)	10,043 (49.2)	10,352 (49.2)	10,394 (49.4)	10,686 (49.5)	96,827 (49.4)
Females	7,812 (50.5)	8,740 (50.4)	8,747 (50.4)	8,713 (50.4)	10,636 (50.3)	11,816 (50.9)	10,387 (50.8)	10,693 (50.8)	10,637 (50.6)	10,917 (50.5)	99,099 (50.6)
**Age**											
15–29 years	4,076 (26.8)	4,337 (25.5)	4,443 (26.0)	4,435(26.0)	5,356 (25.7)	5,602 (24.4)	5,033 (25.2)	5,137 (24.7)	5,205 (25.1)	5,226 (24.5)	48,851 (24.5)
30–49 years	5,838 (38.3)	6,710 (39.4)	6,543 (38.3)	6,512 (38.2)	7,940 (38.1)	8,790 (38.4)	7,456 (37.3)	7,660 (36.9)	7,490 (36.1)	7,781 (36.5)	72,721 (36.5)
50+ years	5,318 (34.9)	5,965 (35.1)	6,087 (35.7)	6 080 (35.7)	7,549 (36.2)	8,523 (37.2)	7,517 (37.6)	7,980 (38.4)	8,043 (38.8)	8,290 (38.9)	71,325 (37.1)
**Education**											
High (University)	3,087 (20.2)	3,803 (22.3)	3,855(22.5)	4,214 (24.8)	5,492 (26.4)	5,309 (23.1)	5,295 (26.5)	5,128 (25.2)	5,426 (26.6)	5,977 (28.4)	47,586 (24.3)
Medium (High school/TAFE)	7,064 (46.2)	7,571 (44.4)	7,740 (45.1)	7,497 (44.2)	9,310 (44.8)	10,639 (46.3)	9,286 (46.5)	9,299 (45.7)	8,996 (44.1)	8,929 (42.4)	86,330 (44.1)
Low (<High school)	5,127 (33.6)	5,689 (33.3)	5,557 (32.4)	5,260 (31.0)	5,988 (28.8)	7,040 (30.6)	5,374 (26.9)	5,923 (29.1)	5,961 (29.2)	6,163 (29.3)	58,082 (29.6)
**IRSAD**^**b**^											
1^st^ (most disadvantaged)	2,536 (16.4)	2,862 (16.6)	2,809,(16.3)	2,752 (15.9)	3,499 (16.6)	3,758 (16.2)	3,246 (16.0)	3,951 (18.8)	3,725 (17.7)	3,911 (18.1)	33,047 (16.9)
2^nd^	3,229 (20.9)	3,603 (20.9)	3,634 (21.1)	3,617 (20.9)	4,183 (19.8)	4,687 (20.2)	4,190 (20.6)	4,589 (21.8)	4,673 (22.2)	4,780 (22.1)	41,185 (21.0)
3^rd^	2,902 (18.8)	3,401 (19.7)	3,338 (19.3)	3,327 (19.3)	3,877 (18.4)	4,773 (20.6)	4,080 (20.1)	4,329 (20.6)	4,234 (20.1)	4,368 (20.2)	38,630 (19.4)
4^th^	3,000 (19.4)	3,240 (18.8)	3,287 (19.0)	3,286 (19.0)	4,238 (20.1)	4,576 (19.8)	3,941 (19.4)	4,006 (19.0)	4,102 (19.5)	3,929 (18.2)	37,606 (19.2)
5^th^ (least disadvantaged)	3,760 (24.4)	4,160 (24.1)	4,188 (24.3)	4,295(24.9)	5,282 (25.1)	5,371 (23.2)	4,873 (24.0)	4,170 (19.8)	4,298 (20.4)	4,615 (21.4)	45,013 (23.0)
**State or territory**											
New South Wales	5,222 (33.7)	5,831 (33.7)	5,810 (33.5)	5,763 (33.3)	7,007 (33.1)	7,664 (33.0)	6,712 (32.9)	6,885 (32.7)	6,842 (32.5)	6,998 (32.4)	64,734 (33.0)
Australian Capital Territory	251 (1.6)	276 (1.6)	274 (1.6)	276 (1.6)	334 (1.6)	375 (1.6)	331 (1.6)	341 (1.6)	338 (1.6)	346 (1.6)	3,142 (1.6)
Northern Territory	144 (0.9)	160 (0.9)	157 (0.9)	153 (0.9)	183 (0.9)	215 (0.9)	191 (0.9)	198 (0.9)	202 (1.0)	210 (1.0)	1,814 (0.9)
Queensland	2,865 (18.5)	3,232 (18.7)	3,270 (18.9)	3,319 (19.2)	4,112 (19.4)	4,528 (19.5)	4,011 (19.6)	4,159 (19.8)	4,187 (19.9)	4,325 (20.0)	38,008 (19.4)
South Australia	1,215 (7.9)	1,351 (7.8)	1,340 (7.7)	1,326 (7.7)	1,611 (7.6)	1,786 (7.7)	1,563 (7.6)	1,602 (7.6)	1,586 (7.5)	1,619 (7.5)	14,998 (7.7)
Tasmania	373 (2.4)	413 (2.4)	412 (2.4)	409 (2.4)	500 (2.4)	549 (2.4)	479 (2.3)	489 (2.3)	486 (2.3)	495 (2.3)	4,604 (2.3)
Victoria	3,893 (25.2)	4,361 (25.2)	4,367 (25.2)	4,346 (25.1)	5,309 (25.1)	5,825 (25.1)	5,122 (25.1)	5,275 (25.1)	5,256 (25.0)	5,399 (25.0)	49,154 (25.1)
Western Australia	1,514 (9.8)	1,701 (9.8)	1,710 (9.9)	1,707 (9.9)	2,093 (9.9)	2,283 (9.8)	2, 022 (9.9)	2,096 (10.0)	2,134 (10.1)	2,211 (10.2)	19,472 (9.9)
**Area of residence**											
Metropolitan	10,693 (69.2)	11,904 (68.8)	11,941 (69.1)	11,857 (68.5)	14,655(69.4)	16,080 (69.3)	14,156 (69.4)	13,975 (66.4)	14,332 (68.1)	14,385 (66.6)	133,977 (68.4)
Inner regional	3,003 (19.4)	3,448 (19.9)	3,384 (19.6)	3,501 (20.2)	4,001 (19.0)	4,598 (19.8)	4,009 (19.7)	4,244 (20.2)	4,152 (19.7)	4,429 (20.5)	38,767 (19.8)
Outer regional/remote	1,756 (11.4)	1,948 (11.3)	1,953 (11.3)	1,940 (11.2)	2449 (11.6)	2,517 (10.9)	2,223 (10.9)	2,827 (13.4)	2,547 (12.1)	2,790 (12.9)	22,950 (11.7)

^a^Weighted estimates for both sample size and percentage

^b^IRSAD: Australian Bureau of Statistics: ‘Index of Relative Socio-Economic Advantage and Disadvantage’ [27].

### Statistical analysis

Analyses were conducted using the Complex Samples module of SPSS version 22 (SPSS Inc. an IBM Company, Chicago, IL). All data were weighted by state, region (metropolitan or rest of the state), age group, gender and year to allow for valid population estimates. The weights were based on Australian Bureau of Statistics (ABS) projections for persons in Occupied Private Dwellings [20]. Pooled prevalence levels and their 95% confidence intervals (95% CI) were calculated for the 12-month recall (2001–10) and for the 2-week recall (2005–10). The prevalence rates and their 95% CIs were also calculated for each year separately, and reported for the total sample and by sociodemographic characteristics. Trends in the prevalence rates over time were examined using linear regression analysis. Differences between the prevalence rates across sociodemographic characteristics were tested using the chi-square test.

Two separate multiple logistic regression analyses were used to assess the odds of being classified as doing ‘sufficient muscle-strengthening activity in the past 12 months (yes/no)’ or ‘sufficient muscle-strengthening activity in the past 2 weeks (yes/no)’. Each model included the following explanatory variables: gender (reference group [ref] = “male”); age (ref = “15–29 years”); education level (ref = “high”); IRSAD (ref = “1^st^ [most disadvantaged]”); State or territory (ref = “New South Wales”) and Area of residence (ref = “metropolitan”). To adjust for yearly variations, year of study was included as a covariate. Adjusted odds ratios and their 95% CIs were reported. Where suitable, the odds ratios were tested for heterogeneity and linear trend using Wald chi-square test and polynomial contrasts method, respectively. A p-value of <0.05 was used to indicate statistical significance.

## Results

The sample sociodemographic characteristics for each year (2001–10) and the pooled weighted data are shown in Table 1. Overall, data were available from 195,926 survey participants (mean yearly sample size = 19,592, range: 15,477–23,226). In the pooled weighted 2001–10 sample, 50.6% were female, 37.1% were aged 50+ years, 68.4% were high school or university educated, 42.2% were in two least disadvantaged IRSAD quintiles, 77.5% were from New South Wales, Victoria or Queensland, and 68.4% were from Metropolitan areas (Table 1).

**Fig 1:**
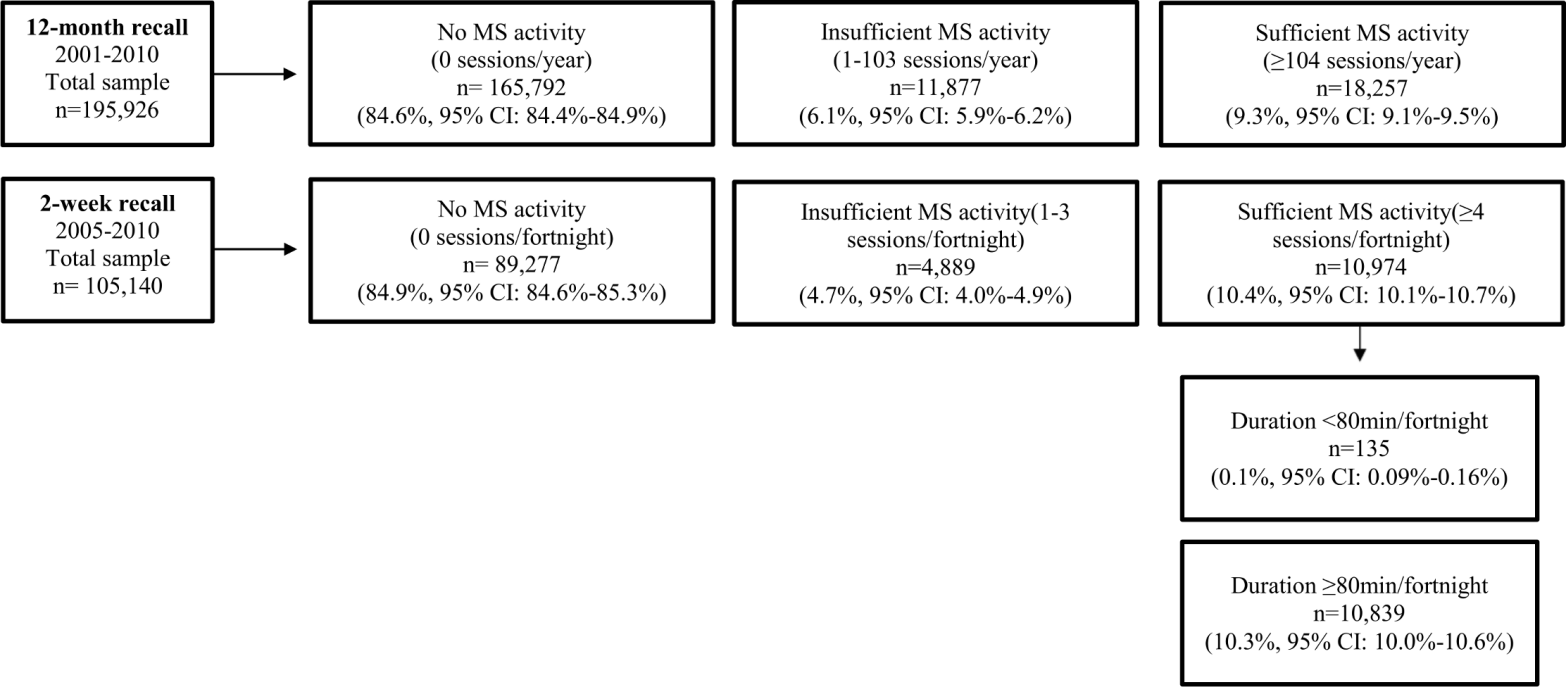
Overview of Exercise, Recreation and Sport Survey (ERASS) responses for muscle-strengthening (MS) activity^a^ prevalence levels reported by weighted counts and percentages (%) and their 95% confidence intervals (95% CI)—pooled responses for 2001–2010 (12-month recall) & 2005–10 (2-week recall). ^a^Muscle-strengthening activities include combined activities assessed within the Exercise, Recreation and Sport Survey (ERASS) 2001–10: Calisthenics, Gymnasium workouts, Military exercise, Prime movers (over 50s), Body building, Circuits, Power team, Weight training for fitness, & Weightlifting (for competition) [20].

The weighted responses for muscle-strengthening activity levels of the pooled ERASS 2001–10 samples are shown in Fig 1. For the 12-month recall (2001–10), 84.6% (95% CI: 84.4–84.9), 6.1% (95% CI: 5.9–6.2), and 9.3% (95% CI: 9.1–9.5) reported no, insufficient and sufficient muscle-strengthening activity, respectively. For the 2-week recall (2005–10), 84.9% (95% CI: 84.6–85.3), 4.7% (95% CI: 4.4–4.9), 10.4% (95% CI: 10.1–10.7) reported no, insufficient frequency of muscle-strengthening activity and sufficient frequency of muscle-strengthening activity, respectively (Fig 1). Almost all (99.9%) of those reporting sufficient frequency of muscle-strengthening activity also reported sufficient duration (Fig 1).

The trends in the prevalence of sufficient muscle-strengthening activity by sociodemographic categories for the 12-month (2001–10), and 2-week recall periods (2005–10) are presented in Tables 2 and 3, respectively. For the total sample, the weighted proportions of those reporting sufficient muscle-strengthening activity showed a significant linear trend over time (p <0.001). The prevalence rates based on 12-month and 2-week recalls increased from 6.4% in 2001 to 12.0% in 2010 and from 9.0% in 2005 to 10.4% in 2010, respectively. Significant linear trends were observed across all sociodemographic categories for the 12-month recall period (2001–10) (p <0.001 for almost all comparisons), and across most sociodemographic categories for the 2-week recall period (p <0.05).

**Table 2:** Proportions (weighted) of Exercise, Recreation and Sport Survey (ERASS) 2001–10 sample reporting sufficient muscle-strengthening activity^a^ during the past 12-months–overall and by selected sociodemographic characteristics.

	Sufficient muscle-strengthening activity in the past 12 months^b^											
	2001	2002	2003	2004	2005	2006	2007	2008	2009	2010	Pooled (2001–10)	
	% (95% CI)											*trend p*^*c*^
**All**	6.4 (5.9–7.0)	7.0 (6.4–7.6)	7.7 (7.1–8.3)	7.8 (7.1–8.4)	8.8 (8.2–9.5)	8.8 (8.2–9.5)	9.8 (9.2–10.4)	11.5 (10.8–12.1)	11.6 (11.0–12.3)	12.0 (11.3–12.6)	9.3 (9.1–9.5)	*<0*.*001*
**Gender**												
Males	6.5 (5.6–7.3)	7.3 (6.4–8.2)	7.8 (6.9–8.7)	7.9 (7.0–8.8)	8.5 (7.6–9.5)	8.3 (7.3–9.2)	8.8 (8.0–9.7)	10.9 (10.0–11.9)	11.1 (10.1–12.1)	11.8 (10.8–12.8)	9.0 (8.7–9.3)	*<0*.*001*
Females	6.4 (2.7–4.0)	6.6 (5.9–7.4)	7.6 (6.8–8.4)	7.6 (6.8–8.5)	9.1 (8.3–10.0)	9.4 (8.5–10.3)	10.8 (10.0–11.6)	12.0 (11.1–12.8)	12.2 (11.3–13.1)	12.1 (11.2–13.0)	9.6 (9.3–9.9)	*<0*.*001*
*p-value*^*d*^	*0*.*894*	*0*.*238*	*0*.*773*	*0*.*661*	*0*.*359*	*0*.*094*	*0*.*001*	*0*.*107*	*0*.*101*	*0*.*655*	0.005	
**Age**												
15-29yrs	9.4 (8.1–10.8)	9.7 (8.2–11.1)	10.5 (9.1–11.9)	12.0 (10.3–13.6)	13.4 (11.7–15.1)	13.0 (11.2–14.8)	12.0 (10.5–13.5)	16.3 (14.6–18.0)	15.9 (14.0–17.7)	15.3 (13.5–17.2)	12.9 (12.4–13.5)	*<0*.*001*
30-49yrs	7.3 (6.3–8.2)	8.5 (7.5–9.6)	8.9 (7.9–9.9)	8.9 (7.8–9.9)	9.8 (8.8–10.9)	9.9 (8.8–11.0)	12.3 (11.3–13.3)	12.6 (11.5–13.6)	13.4 (12.3–14.6)	14.3 (13.1–15.5)	10.7 (10.4–11.1)	*<0*.*001*
50+yrs	3.3 (2.7–4.0)	3.4 (2.8–4.1)	4.6 (3.8–5.3)	3.7 (3.0–4.3)	4.7 (4.0–5.5)	5.1 (4.3–5.8)	6.1 (5.4–6.8)	7.3 (6.6–8.1)	7.3 (6.6–8.0)	7.7 (7.0–8.4)	5.5 (5.3–5.7)	*<0*.*001*
*p-value*^*d*^	*<0*.*001*	*<0*.*001*	*<0*.*001*	*<0*.*001*	*<0*.*001*	*<0*.*001*	*<0*.*001*	*<0*.*001*	*<0*.*001*	*<0*.*001*	*<0*.*001*	
**Education**												
High	9.6 (8.1–11.1)	10.5 (9.0–12.0)	10.7 (9.3–12.2)	11.7 (10.2–13.3)	11.4 (10.-12.8)	11.9 (10.3–13.5)	13.2 (11.9–14.5)	15.2 (13.8–16.7)	15.3 (13.8–16.8)	15.7 (14.3–17.1)	12.8 (12.4–13.3)	*<0*.*001*
Medium	7.5 (6.6–8.4)	7.7 (6.8–8.6)	8.4 (7.4–9.3)	8.2 (7.2–9.1)	9.5 (8.5–10.5)	9.1 (8.1–10.1)	9.6 (8.8–10.5)	11.6 (10.6–12.6)	12.5 (11.4–13.5)	13.4 (12.2–14.5)	9.9 (9.5–10.2)	*<0*.*001*
Low	3.2 (2.6–3.9)	3.7 (3.0–4.5)	4.9 (4.1–5.7)	4.2 (3.3–5.0)	5.9 (4.9–6.8)	6.2 (5.2–7.2)	6.8 (5.8–7.8)	8.3 (7.2–9.4)	7.5 (6.5–8.5)	6.9 (5.9–7.9)	5.8 (5.5–6.1)	*<0*.*001*
*p-value*^*d*^	*<0*.*001*	*<0*.*001*	*<0*.*001*	*<0*.*001*	*<0*.*001*	*<0*.*001*	*<0*.*001*	*<0*.*001*	*<0*.*001*	*<0*.*001*	*<0*.*001*	
**IRSAD**												
1st (most)	4.2 (3.2–5.3)	5.2 (4.0–6.5)	5.4 (4.2–6.6)	4.8 (3.7–6.0)	5.1 (3.9–6.2)	3.3 (4.9–7.6)	7.5 (6.2–8.9)	8.7 (7.3–10.0)	8.3 (6.9–9.7)	7.9 (6.7–9.2)	6.5 (6.1–6.9)	*<0*.*001*
2^nd^	5.4 (4.3–6.5)	4.6 (3.6–5.6)	5.6 (4.6–6.7)	6.6 (5.4–7.8)	5.8 (4.7–6.9)	6.8 (5.6–8.0)	7.8 (6.6–9.0)	9.9 (8.5–11.2)	10.7 (9.3–12.1)	10.7 (9.3–12.1)	7.6 (7.2–8.0)	*<0*.*001*
3^rd^	5.8 (4.6–7.0)	7.1 (5.8–8.4)	7.7 (6.3–9.1)	7.2 (5.9–8.6)	8.6 (7.1–10.1)	9.2 (7.7–10.7)	9.4 (8.2–10.7)	11.1 (9.7–12.6)	11.7 (10.2–13.2)	11.2 (9.7–12.7)	9.1 (8.7–9.6)	*<0*.*001*
4^th^	7.1 (5.8–8.4)	7.5 (6.1–8.9)	9.2 (7.7–10.7)	8.2 (6.7–9.6)	12.0 (10.4–13.7)	9.7 (8.1–11.2)	10.7 (9.4–12.1)	13.8 (12.2–15.3)	12.1 (10.7–13.6)	13.8 (12.2–15.4)	10.6 (10.1–12.5)	*<0*.*001*
5th (least)	8.8 (7.4–10.1)	9.8 (8.4–11.3)	10.1 (8.6–11.5)	10.8 (9.3–12.3)	11.2 (9.7–12.7)	11.5 (9.9–13.1)	12.8 (11.4–14.2)	14.0 (12.4–15.5)	15.0 (13.3–16.6)	15.8 (14.1–17.4)	12.0 (11.5–12.5)	*<0*.*001*
*p-value*^*d*^	*<0*.*001*	*<0*.*001*	*<0*.*001*	*<0*.*001*	*<0*.*001*	*<0*.*001*	*<0*.*001*	*<0*.*001*	*<0*.*001*	*<0*.*001*	*<0*.*001*	
**State**^**e**^												
NSW	6.0 (4.9–7.1)	6.2 (5.1–7.3)	7.5 (6.3–8.7)	7.6 (6.3–8.8)	8.7 (7.4–10.0)	8.8 (7.5–10.1)	9.4 (8.1–10.6)	10.7 (9.2–12.1)	11.3 (9.8–12.8)	11.0 (9.5–12.4)	8.9 (8.4–9.3)	*<0*.*001*
ACT	8.3 (4.9–7.1)	8.7 (7.3–10.1)	11.1 (9.6–12.7)	12.5 (10.9–14.1)	11.6 (10.0–13.3)	11.8 (10.2–13.5)	13.1 (11.3–14.8)	15.0 (13.2–16.9)	15.1 (13.2–17.0)	14.7 (12.8–16.5)	12.4 (11.9–12.9)	*<0*.*001*
NT	6.7 (5.0–8.3)	9.0 (7.0–10.9)	7.3 (5.7–8.9)	8.3 (6.5–10.1)	6.9 (5.4–8.4)	8.4 (6.7–10.2)	9.5 (7.8–11.3)	11.9 (9.8–13.9)	10.4 (8.4–12.4)	11.2 (9.2–13.3)	9.1 (8.5–9.7)	*0*.*005*
QLD	6.4 (5.1–7.7)	7.3 (6.0–8.6)	6.4 (5.1–7.6)	6.3 (5.0–7.5)	9.8 (8.2–11.3)	8.2 (6.8–9.6)	10.4 (8.9–12.0)	11.3 (9.6–12.9)	11.0 (9.4–12.7)	13.0 (11.2–14.7)	9.3 (8.8–9.7)	*<0*.*001*
SA	5.5 (4.3–6.7)	6.9 (5.6–8.2)	7.7 (6.3–9.0)	6.8 (5.5–8.1)	6.5 (5.2–7.8)	7.9 (6.4–9.3)	9.0 (7.6–10.5)	13.0 (11.2–14.8)	10.6 (8.9–12.3)	12.5 (10.7–14.4)	8.8 (8.3–9.3)	*0*.*001*
TAS	5.5 (4.3–6.6)	5.2 (4.0–6.3)	5.9 (4.8–7.1)	6.7 (5.4–7.9)	5.4 (4.2–6.6)	5.6 (4.4–6.8)	7.7 (6.3–9.1)	8.1 (6.6–9.5)	6.9 (5.5–8.2)	8.0 (6.5–9.4)	6.5 (6.1–6.9)	*0*.*006*
VIC	7.1 (5.9–8.3)	7.2 (5.9–8.5)	8.3 (6.9–9.6)	9.0 (7.5–10.4)	8.8 (7.5–10.2)	9.4 (7.9–10.9)	9.7 (8.8–10.6)	12.5 (11.5–13.4)	12.2 (11.3–13.2)	12.3 (11.3–13.2)	9.8 (9.4–10.2)	*<0*.*001*
WA	6.8 (5.6–8.1)	8.6 (7.2–10.0)	9.6 (8.1–11.1)	8.5 (7.1–10.0)	9.7 (8.3–11.2)	9.8 (8.2–11.5)	11.2 (9.6–12.7)	11.0 (9.4–12.6)	13.8 (12.0–15.7)	12.4 (10.6–14.2)	10.3 (9.8–10.9)	*<0*.*001*
*p-value*^*d*^	*0*.*364*	*0*.*082*	*0*.*012*	*0*.*005*	*0*.*022*	*0*.*141*	*0*.*109*	*0*.*027*	*0*.*009*	*0*.*055*	*<0*.*001*	
**Area**												
Metro	7.4 (6.6–8.1)	8.2 (7.4–9.0)	8.9 (8.1–9.7)	9.2 (8.3–10.0)	10.0 (9.2–10.9)	10.3 (9.5–11.2)	12.2 (10.4–12.0)	12.7 (11.9–13.5)	13.4 (12.5–14.3)	13.8 (12.9–14.7)	10.7 (10.4–11.0)	*<0*.*001*
Inner	4.6 (3.6–5.5)	4.6 (3.7–5.6)	5.2 (4.1–6.2)	4.9 (3.9–5.9)	6.8 (5.6–8.0)	5.6 (4.5–6.7)	7.3 (6.1–8.4)	10.1 (8.6–11.7)	8.0 (6.9–9.2)	8.7 (7.4–10.0)	6.7 (6.4–7.1)	*0*.*001*
Remote	4.0 (2.9–5.1)	3.5 (2.6–4.4)	5.0 (3.9–6.2)	4.3 (3.2–5.4)	4.8 (3.7–6.0)	5.2 (3.9–6.5)	5.9 (4.6–7.3)	7.2 (5.9–8.6)	7.3 (6.1–8.4)	7.8 (6.8–14.7)	*5*.*7 (5*.*3–6*.*1)*	*<0*.*001*
*p-value*^*d*^	*<0*.*001*	*<0*.*001*	*<0*.*001*	*<0*.*001*	*<0*.*001*	*<0*.*001*	*<0*.*001*	*<0*.*001*	*<0*.*001*	*<0*.*001*	*<0*.*001*	

^a^Muscle-strengthening activities include combined activities assessed within the Exercise, Recreation and Sport Survey (ERASS) 2001–10: Calisthenics, Gymnasium workouts, Military exercise, Prime movers (over 50s), Body building, Circuits, Power team, Weight training for fitness, & Weightlifting (for competition) [20].

^b^Prevalence of respondents who reported participating in at least 104 sessions of muscle-strengthening activity in the past 12 months.

^c^p-value for linear regression analysis of the proportion over time (2001–2010).

^d^p-value for chi-square test of the difference between sociodemographic categories.

^e^NSW = New South Wales, ACT = Australian Capital Territory, NT = Northern Territory, QLD = Queensland, SA = South Australia, TAS = Tasmania, VIC = Victoria, WA = Western Australia.

**Table 3:** Proportions (weighted) of Exercise, Recreation and Sport Survey (ERASS) 2005–10 sample reporting sufficient frequency of muscle-strengthening activity^a^ during the past 2 weeks–overall and by selected sociodemographic characteristics.

	Sufficient muscle-strengthening activity in the past 2 weeks^b^							
	2005	2006	2007	2008	2009	2010	Pooled (2005–10)	
	% (95% CI)							*trend p*^*c*^
**All**	9.0 (8.3–9.7)	9.8 (9.0–10.5)	10.1 (9.4–10.8)	10.9 (10.2–11.6)	11.1 (10.4–11.9)	11.8 (11.0–12.5)	10.4 (10.1–10.7)	*<0*.*001*
**Gender**								
Males	9.0 (7.9–10.1)	9.8 (9.0–10.5)	9.5 (8.5–10.5)	11.0 (10.0–12.1)	10.9 (9.9–12.0)	11.8 (10.7–12.9)	10.4 (10.0–10.9)	*0*.*012*
Females	9.1 (8.1–10.0)	11.1 (9.9–12.2)	10.7 (9.7–11.6)	10.7 (9.8–11.6)	11.4 (10.4–12.3)	11.7 (10.7–12.8)	10.4 (10.1–10.8)	*<0*.*001*
*p-value*^*d*^	*0*.*931*	*0*.*248*	*0*.*098*	*0*.*618*	*0*.*583*	*0*.*962*	*0*.*949*	
**Age**								
15–29 years	12.3 (10.5–14.1)	13.1 (11.2–15.0)	11.9 (10.3–13.5)	14.2 (12.5–16.0)	14.5 (12.6–16.4)	14.9 (13.0–16.9)	13.5 (12.8–14.2)	*0*.*033*
30–49 years	9.8 (8.7–10.9)	10.9 (9.6–12.2)	11.1 (10.1–12.2)	11.9 (10.8–13.1)	12.2 (11.1–13.4)	13.3 (12.0–14.5)	11.5 (11.0–12.0)	*<0*.*001*
50+ years	5.6 (4.7–6.5)	5.9 (5.0–6.8)	7.7 (6.8–8.7)	7.4 (6.5–8.2)	7.7 (6.8–8.5)	7.9 (7.1–8.7)	7.0 (6.7–7.4)	*0*.*024*
*p-value*^*d*^	*<0*.*001*	*<0*.*001*	*<0*.*001*	*<0*.*001*	*<0*.*001*	*<0*.*001*	*<0*.*001*	
**Education**								
High (University)	11.0 (9.5–12.5)	12.3 (10.6–14.0)	12.6 (11.2–14.0)	13.8 (12.3–15.2)	14.4 (12.8–15.9)	14.4 (13.0–15.9)	13.1 (12.5–13.7)	*0*.*002*
Medium (High school/TAFE)	9.3 (8.2–10.4)	10.1 (8.9–11.2)	9.7 (8.8–10.7)	10.9 (9.8–12.0)	11.0 (9.9–12.1)	13.0 (11.7–14.2)	10.7 (10.2–11.1)	*0*.*012*
Low (<High school)	6.4 (5.3–7.6)	6.9 (5.7–8.2)	7.6 (6.4–8.9)	7.9 (6.7–9.1)	8.2 (7.0–9.4)	7.2 (6.1–8.3)	7.4 (6.9–7.8)	*0*.*163*
*p-value*^*d*^	*<0*.*001*	*<0*.*001*	*<0*.*001*	*<0*.*001*	*<0*.*001*	*<0*.*001*	*<0*.*001*	
**IRSAD**								
1st (most disadvantaged)	5.7 (4.3–7.1)	7.5 (5.7–9.2)	9.0 (7.2–10.7)	8.6 (7.1–10.1)	8.5 (6.9–10.2)	8.2 (6.7–9.7)	7.9 (7.3–8.6)	*0*.*128*
2^nd^	6.0 (4.7–7.3)	8.3 (6.7–9.8)	8.4 (7.0–9.8)	9.6 (8.1–11.1)	10.0 (8.5–11.4)	10.7 (9.0–12.3)	8.9 (8.3–9.5)	*0*.*003*
3^rd^	9.3 (7.6–10.9)	9.9 (8.2–11.6)	8.7 (7.4–10.0)	9.9 (8.4–11.3)	11.6 (9.9–13.2)	11.7 (10.0–13.4)	10.2 (9.5–10.8)	*0*.*055*
4^th^	11.7 (10.0–13.5)	10.0 (8.3–11.7)	11.7 (10.1–13.2)	12.5 (10.9–14.1)	10.8 (9.2–12.3)	13.0 (11.3–14.8)	11.6 (10.9–12.3)	*0*.*352*
5th (least disadvantaged)	10.6 (9.1–12.2)	12.0 (10.2–13.7)	12.1 (10.6–13.6)	13.4 (11.7–15.0)	14.3 (12.5–16.0)	14.3 (12.6–16.1)	12.7 (12.0–13.4)	*0*.*001*
*p-value*^*d*^	*<0*.*001*	*<0*.*001*	*<0*.*001*	*<0*.*001*	*<0*.*001*	*<0*.*001*	*<0*.*001*	
**State or territory**								
New South Wales	9.4 (7.9–10.8)	9.8 (8.3–11.3)	9.5 (8.1–11.0)	11.0 (9.4–12.5)	11.1 (9.5–12.8)	11.7 (10.0–13.4)	10.4 (9.8–11.0)	*0*.*005*
Australian Capital Territory	11.2 (9.4–13.0)	13.0 (11.1–14.9)	12.6 (10.8–14.4)	12.1 (10.4–13.9)	14.3 (12.4–16.3)	13.5 (11.6–15.4)	12.8 (12.0–13.6)	*0*.*095*
Northern Territory	6.7 (5.1–8.3)	9.1 (7.1–11.1)	10.5 (8.4–12.6)	10.6 (8.5–12.8)	10.0 (7.8–12.2)	11.7 (9.4–14.1)	9.8 (9.0–10.7)	*0*.*026*
Queensland	10.3 (8.6–12.0)	8.9 (7.3–10.6)	10.8 (9.0–12.5)	10.6 (8.8–12.3)	11.0 (9.3–12.8)	12.2 (10.3–14.1)	10.6 (9.9–11.3)	*0*.*064*
South Australia	6.4 (5.0–7.8)	8.3 (6.6–9.9)	10.1 (8.4–11.8)	11.3 (9.5–13.1)	8.7 (7.0–10.4)	13.5 (11.4–15.6)	9.7 (9.0–10.4)	*0*.*047*
Tasmania	5.1 (3.8–6.4)	5.5 (4.1–6.8)	7.8 (6.2–9.3)	7.3 (5.7–8.9)	7.1 (5.5–8.6)	7.8 (6.2–9.4)	6.7 (6.1–7.3)	*0*.*050*
Victoria	8.6 (7.2–10.1)	10.9 (9.1–12.6)	9.9 (8.8–10.9)	11.3 (10.3–12.2)	11.9 (10.9–13.0)	11.5 (10.4–12.6)	10.7 (10.2–11.2)	*0*.*043*
Western Australia	9.3 (7.7–10.8)	10.0 (8.2–11.8)	11.2 (9.5–12.9)	10.4 (8.7–12.1)	11.7 (9.8–13.6)	11.2 (9.3–13.1)	10.6 (9.9–11.4)	*0*.*047*
*p-value*^*d*^	*0*.*019*	*0*.*062*	*0*.*228*	*0*.*493*	*0*.*056*	*0*.*284*	*<0*.*001*	
**Area of residence**								
Metropolitan	10.2 (9.2–11.1)	11.1 (10.1–12.1)	11.2 (10.4–12.1)	12.2 (11.3–13.1)	12.7 (11.7–13.6)	13.3 (12.3–14.3)	11.8 (11.4–12.1)	*<0*.*001*
Inner regional	6.7 (5.4–8.0)	7.0 (5.6–8.3)	7.8 (6.5–9.1)	9.5 (7.9–11.1)	8.0 (6.8–9.3)	9.3 (7.8–10.8)	5.4 (5.0–5.8)	*0*.*041*
Outer regional/remote	5.2 (3.8–6.6)	5.9 (4.3–7.5)	7.1 (5.4–8.8)	5.7 (4.4–7.0)	7.3 (6.1–8.6)	7.5 (6.4–8.7)	6.5 (5.9–7.0)	*0*.*058*
*p-value*^*d*^	*<0*.*001*	*<0*.*001*	*<0*.*001*	*<0*.*001*	*<0*.*001*	*<0*.*001*	*<0*.*001*	

^a^Muscle-strengthening activities include combined activities assessed within the Exercise, Recreation and Sport Survey (ERASS) 2001–10: Calisthenics, Gymnasium workouts, Military exercise, Prime movers (over 50s), Body building, Circuits, Power team, Weight training for fitness, & Weightlifting (for competition) [20].

^b^Prevalence of respondents who reported participating in at least 4 muscle-strengthening activity sessions in the past 2 weeks.

^c^p-value for linear regression trend between 2005–10.

^d^p-value for chi-square test of the difference between sociodemographic categories.

Table 4 shows the results of the multiple logistic regression analyses. For most sociodemographic categories, the adjusted odds ratios of reporting sufficient muscle-strengthening activity were largely concordant for both recall periods. When compared to reference groups, older adults (p <0.001) those with lower education (p <0.001) and those living in non-metropolitan areas (p<0.001) were significantly less likely to report sufficient muscle-strengthening activity. The odds of sufficient muscle-strengthening activity increased with higher socioeconomic status (p-value for trend <0.001 for both recall periods). In addition, females were somewhat more likely than males to report sufficient muscle-strengthening activity in the past 12 months, but not in the past 2 weeks (Table 4).

**Table 4:** Adjusted odds ratios^a^ (OR) (weighted), and their 95% confidence intervals (95% CI), for participation in ‘Sufficient muscle-strengthening activity^b^‘ in the past 12 months, and in the past 2 weeks.

Explanatory variable	Sufficient muscle-strengthening activity in the past 12 months^c^	Sufficient muscle-strengthening activity in the past 2 weeks^d^
	OR (95% CI)	OR (95% CI)
**Sex (ref; male)**		
Females	1.09 (1.04–1.15)	1.02 (0.95–1.09)
*p-value*	*<0*.*001*	0.576
**Age (ref; 15–29 years)**		
30–49 years	0.73 (0.69–0.78)	0.75 (0.69–0.82)
50+ years	0.40 (0.37–0.42)	0.48 (0.44–0.52)
*p [heterogeneity]*^*e*^	*<0*.*001*	*<0*.*001*
*p [trend]*^*f*^	*<0*.*001*	*<0*.*001*
**Education (ref; high [university])**		
Medium (High school/TAFE)	0.80 (0.76–0.85)	0.82 (0.76–0.88)
Low (<High school)	0.49 (0.46–0.53)	0.56 (0.51–0.62)
*p [heterogeneity]*^*e*^	*<0*.*001*	*<0*.*001*
*p [trend]*^*f*^	*<0*.*001*	*<0*.*001*
**SEIFA (ref; 1st [most disadvantaged])**		
2nd	1.04 (0.95–1.14)	1.02 (0.90–1.15)
3rd	1.13 (1.03–1.24)	1.06 (0.93–1.20)
4th	1.19 (1.08–1.31)	1.11 (0.98–1.26)
5th (least disadvantaged)	1.32 (1.20–1.46)	1.16 (1.20–1.33)
*p [heterogeneity]*^*e*^	*<0*.*001*	*0*.*095*
*p [trend]*^*f*^	*<0*.*001*	*<0*.*001*
**State or territory (ref; New South Wales)**		
Australian Capital Territory	1.10 (1.02–1.19)	1.02 (0.92–1.14)
Northern Territory	1.56 (1.37–1.78)	1.49 (1.25–1.77)
Queensland	1.16 (1.07–1.26)	1.12 (1.00–1.24)
South Australia	1.12 (1.03–1.21)	1.00 (0.80–1.05)
Tasmania	1.13 (1.02–1.26)	0.92 (0.79–1.26)
Victoria	1.12 (1.05–1.20)	1.02 (0.94–1.12)
Western Australia	1.21 (1.12–1.31)	1.05 (0.94–1.17)
*p [heterogeneity]*^*e*^	*<0*.*001*	*<0*.*001*
**Area of residence (ref; metropolitan)**		
Inner regional	0.76 (0.70–0.82)	0.77 (0.70–0.86)
Outer regional/remote	0.59 (0.53–0.65)	0.57 (0.50–0.65)
*p [heterogeneity]*^*e*^	*<0*.*001*	*<0*.*001*

^a^Adjusted for year and all other explanatory variables in the table.

^b^Muscle-strengthening activities include combined activities assessed within the Exercise, Recreation and Sport Survey (ERASS) 2001–10: Calisthenics, Gymnasium workouts, Military exercise, Prime movers (over 50s), Body building, Circuits, Power team, Weight training for fitness, & Weightlifting (for competition) [20].

^c^To be classified as having ‘Sufficient muscle-strengthening activity in the past 12 months’, respondents had to report at least 104 sessions in the past 12 months.

^d^To be classified as having ‘Sufficient muscle-strengthening activity in the past 2 weeks’, respondents had to report at least 4 sessions in the past 2 weeks.

^e^p-value for heterogeneity based on the likelihood ratio Wald chi-square test.

^f^p-value for linear contrast of ordinal categories.

## Discussion

The key finding is that around 90% of Australians do not meet the global and national muscle-strengthening activity recommendations. Given the multiple health benefits associated with participation in sufficient muscle-strengthening activity [10], these findings are of a public health concern. A further key outcome was the approximate two-fold increase in muscle-strengthening activity levels during the ERRAS 2001–10 monitoring period.

Previous smaller Australian studies have estimated that 9.4%-18.6% met the muscle-strengthening activity recommendations [16–18], compared to ~10% in the current study. The differences in prevalence estimates may be due to different sample sizes and survey methodologies. The ERASS reported on muscle-strengthening activity in the past 12-months and 2-weeks, whereas other two studies reported past-week recalls only [17, 18]. Moreover, slight discrepancies in questionnaire items wording may have added to the magnitude of the differences between prevalence rates. This underscores the importance of using standardised instruments in public health surveillance of muscle-strengthening activity. Interestingly, the 12-month and 2-week recall periods produced comparable estimates in the current study.

A potential positive finding is that muscle-strengthening activity levels significantly increased between 2001–10. Since the ERASS study period did not coincide with 2014 Australia's Physical Activity Guidelines, which where the first national guidelines to recommend muscle-strengthening activity, it is unlikely that public health campaigns were responsible. A possible explanation may be the increase in the number of privatized gym facilities in Australia over the last decade [29], hence, potentially providing better access to muscle-strengthening equipment. In addition, there are some data to suggest that more people are transitioning out of organised sport and into less-organised activities [30]. Regardless of these trends, it should be reiterated that even in 2010 as much as 88% of the sample did not report sufficient participation in muscle-strengthening activity.

The sociodemographic correlates of muscle-strengthening activity in the current study are largely consistent with previous research [6]. Both internationally [6] and within Australia [16], previous studies have shown that older adults, those with lower education levels and socioeconomically disadvantaged individuals are less likely to report sufficient muscle-strengthening activity. Our findings support targeting these groups in strategies to increase muscle-strengthening activity levels. Also, it might be possible that encouraging muscle-strengthening activity participation among younger populations may result in higher participation across the lifespan. In the current study, females were more likely than males to report sufficient muscle-strengthening activity in the last 12 months, but not when a 2-week recall was used. Further research is needed to examine the causes of this difference between two recall periods. Nevertheless, a previous Australian study has found slightly higher participation rate among females, however, due to a smaller sample size the difference did not reach the level of statistical significance [25]. It may be that in future Australian interventions to increase muscle-strengthening activity participation more focus should be placed on older males.

When compared to the proportions of Australian adults meeting the moderate-to-vigorous-intensity aerobic activity guidelines [31], fewer the Australian adults meet the muscle-strengthening activity guidelines (~50% vs. ~10%). In fact, the present study suggests that a large majority of Australians that did not meet the muscle-strengthening activity recommendations, are most likely not attaining the numerous health benefits associated with this activity [10].

Our findings call for public health strategies to encourage increasing participation in muscle-strengthening activity [32]. At the macro-level, it is incumbent upon governments to provide supportive policy and physical environments. Strategies could include the provision of affordable and accessible community-based facilities (e.g. gyms/health clubs) and the subsidising of equipment (e.g. resistance bands, dumbbells). Supporting these strategies is emerging evidence from cross-sectional studies in Japan suggesting that key correlates of muscle-strengthening activity participation include perceived access to facilities and the existence of equipment at home [33, 34].

This study has several limitations. First, the ambiguous ERASS coding for some activities, such as ‘gymnasium workouts’, may have captured non-muscle-strengthening-related activities (e.g. aerobic, flexibility training), potentially resulting in an overestimation of muscle-strengthening activity levels. Since only leisure-time physical activity was assessed, a further limitation was that any muscle strengthening-related activities that occurred within the occupational and household domains were not reported (e.g. farming, labouring, gardening etc.). However, given recent technological and societal advancements [35], we believe it is unlikely that many accrue sufficient muscle-strengthening activity within these domains. A further limitation was the fact that the sample included respondents aged 15–18 years. It is likely that such respondents may not have completed their full education, and therefore be classified as having low or medium education levels. Therefore, our findings from unadjusted analyses around the prevalence of muscle-strengthening activity by education level should be viewed with caution. Last, previous studies reporting ERASS data have shown an under-representation of non-English speaking respondents, and therefore results may be less generalizable to non-native speakers [23]. Strengths of this study include the use of large broadly representative national sample [22], and the assessment of muscle-strengthening activity levels over multiple years and across several sociodemographic categories. A further strength was that both the 12-month recall and 2-week recall yielded similar results. This supports the robustness of our finding.

## Conclusion

Between 2001 and 2010 a vast majority of Australians reported no or insufficient participation in muscle-strengthening activity. Given the considerable health benefits associated with participation in this type of physical activity, these findings should prompt public health agencies to formulate comprehensive approaches to support population-level muscle-strengthening activity participation. At risk population sub-groups include older adults, those living in socioeconomically disadvantaged and non-metropolitan areas, and those with low education levels.
